# Dataset on the spent filter backwash water treatment by sedimentation, coagulation and ultra filtration

**DOI:** 10.1016/j.dib.2017.10.062

**Published:** 2017-11-02

**Authors:** Mokhtar Mahdavi, Afshin Ebrahimi, Hossein Azarpira, Hamid Reza Tashauoei, Amir Hossein Mahvi

**Affiliations:** a Environmental Health Engineering Department, Saveh University of Medical Sciences, Social Determinants of Health Research Center, Saveh, Iran; b Department of Environmental Health Engineering, Environment Research Center, Research Institute for Primordial Prevention of Non Communicable Disease, Isfahan University of Medical Sciences, Isfahan, Iran; c Department of Environmental Health Engineering, School of Health, Islamic Azad University Tehran Medical Branch, Tehran, Iran; d School of Public Health, Tehran University of Medical Science, Tehran, Iran; e Center for Solid Waste Research, Institute for Environmental Research, Tehran University of Medical Science, Tehran, Iran

**Keywords:** Spent filter backwash water, Water treatment, Coagulation, Ultra-filtration

## Abstract

During operation of most water treatment plants, spent filter backwash water (SFBW) is generated, which accounts about 2–10% of the total plant production. By increasing world population and water shortage in many countries, SFBW can be used as a permanent water source until the water treatment plant is working. This data article reports the practical method being used for water reuse from SFBW through different method including pre-sedimentation, coagulation and flocculation, second clarification, ultra filtration (UF) and returned settled SFBW to the beginning of water treatment plant (WTP). Also, two coagulants of polyaluminum ferric chloride (PAFCl) and ferric chloride (FeCl_3_) were investigated with respect to their performance on treated SFBW quality. Samples were collected from Isfahan's WTP in Iran during spring and summer season. The acquired data indicated that drinkable water can be produced form SFBW by applying hybrid coagulation-UF process (especially when PAFCl used as coagulant).

**Specifications Table**

**Table:** 

Subject area	Environmental Engineering
More specific subject area	Water treatment, water reuse
Type of data	Table and figure
How data was acquired	Raw SFBW was treated with a pilot plant that includes primary sedimentation, coagulation & flocculation and ultra-filter. The quality of raw water in Isfahan's WTP, produced and treated SFBW was determined according to the standard method for the examination of water and wastewater.
Data format	Raw and analysed
Experimental factors	– The data related to sedimentation, coagulation (with PAFCl and FeCl_3_) and UF was presented – The data related to quality of raw and treated SFBW including biological, chemical and physical properties was presented.
Experimental features	SFBW treatment by primary sedimentation, coagulation and flocculation and ultra-filter
Data source location	Isfahan's WTP in Iran
Data accessibility	The data are available with this article and it is not published anywhere

**Value of the data**

• The data presents the quality of raw water and produced spent filter backwash water in Isfahan- Iran water treatment plant.
• This data show the ability of two coagulant as traditional and pre polymerized for SFBW treatment.
• The data present the quality of treated SFBW with coagulation - UF process and returned the settled SFBW to the WTP entrance.

## 1 Data

**Fig. 1:**
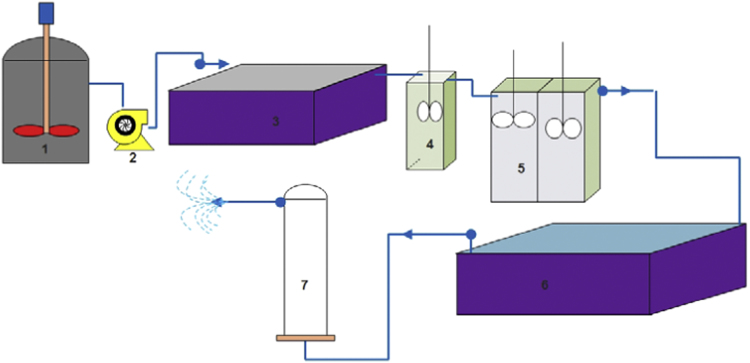
A schematic of the experimental set-up. 1: a reservoir tank for raw SFBW, 2: pump, 3: primary sedimentation, 4: coagulation, 5: flocculation, 6: secondary sedimentation, 7: UF module [6].

**Fig. 2:**
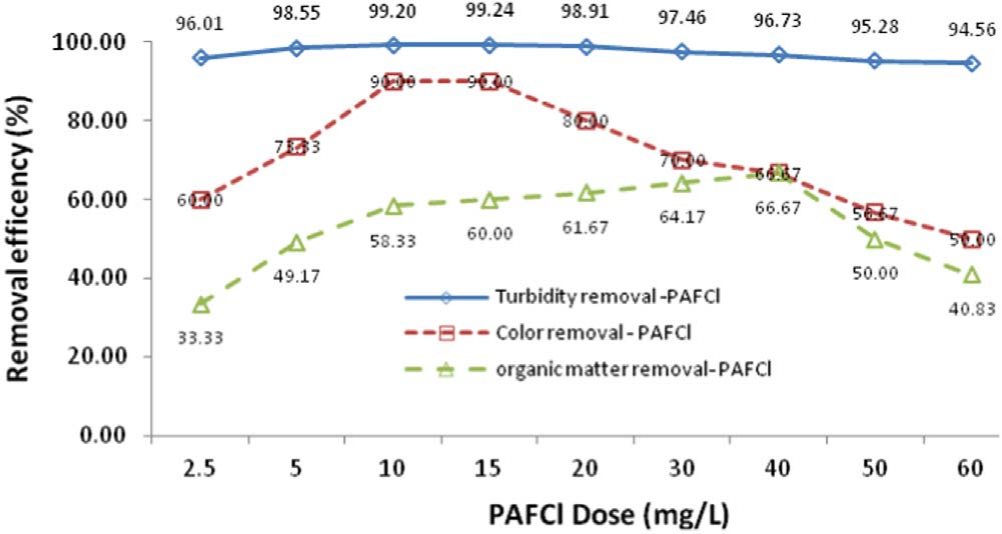
Affect of PAFCl at various doses (from 2.5 to 60 mg/L) on turbidity, color and organic matter removal from SFBW.

**Fig. 3:**
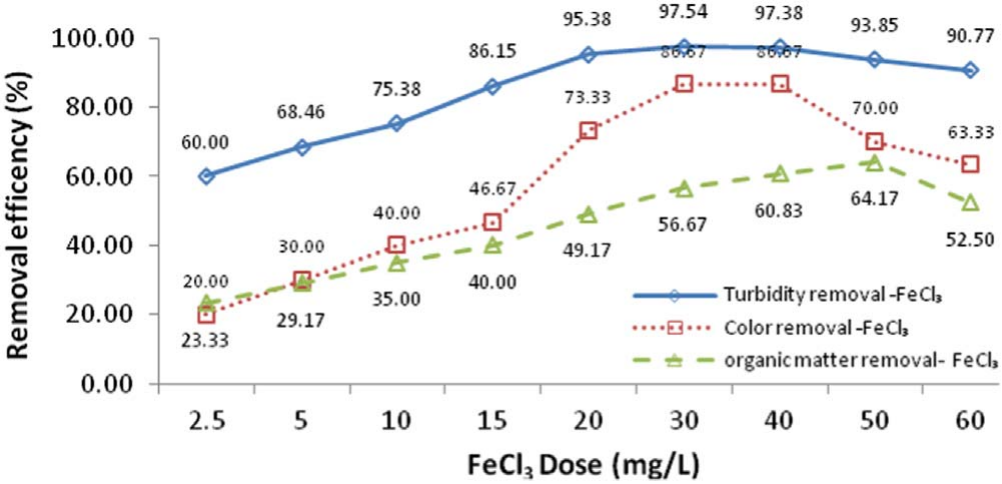
Affect of FeCl_3_ at various doses (from 2.5 to 60 mg/L) on turbidity, color and organic matter removal from SFBW.

Presented data in this article deal with the quality of raw water entered to Isfahan's WTP, raw produced SFBW and treated SFBW by primary sedimentation, coagulation (PAFCl and FeCl_3_ used as coagulants), Hybrid coagulation-UF processes and recirculation of primary settled SFBW to WTP entrance. Data including parameters like turbidity, color, electrical conductivity (EC), total dissolved solid (TDS), pH, alkalinity, Sludge volume, Iron (Fe), Aluminium (Al), Arsenic (As), Lead (Pb), Cadmium (Cd), ultra violet adsorption at 254 nm wave length (UVA_254_), specific ultra violet (SUVA), dissolved organic carbon (DOC), total organic carbon (TOC), total coliform (TC), fecal coliform (FC) and heterotrophic plate count (HPC). These data presented in Table 1, Table 2, Table 3 and Fig. 1, Fig. 2, Fig. 3.

**Table 1:** The quality of raw water entered to WTP, produced water in WTP and SFBW that produced during filters backwash.

Parameter	Raw water in WTP	Produced water in WTP	Raw SFBW
Turbidity (NTU)	7 (± 0.7)	0.25 (± 0.01)	275.5 (± 2.1)
Color (Pt. Co. units)	11 (± 1.4)	0	35 (± 2.8)
EC (μs/cm)	333 (± 2.8)	334 (± 1.4)	335 (± 1.4)
TDS (mg/L)	165 (± 2.8)	166 (± 1.41)	167 (± 1.4)
pH	8.22 (± 0.02)	8.2 (± 0.02)	8.4 (± 0.1)
Alkalinity (mg/L CaCO_3_)	132 (± 1.4)	126 (± 1.4)	150 (± 2.8)
Sludge volume (ml/L)	Negligible	Negligible	12 (± 1.4)
Iron (mg/L)	0.1 (± 0.01)	0.02 (± 0.002)	0.7 (± 0.002)
Aluminium (mg/L)	0.035 (± 0.01)	0.049 (± 0.002)	0.31 (± 0.002)
Arsenic (µ/L)	0	0	0
Lead (µ/L)	2 (± 0.28)	0.5 (± 0.002)	16 (± 0.002)
Cadmium (µ/L)	0.43 (± 0.028)	0.38 (± 0.002)	0.7 (± 0.002)
UVA_254nm_ (cm^-1^)	0.052 (± 0.03)	0.034 (± 0.001)	0.18 (± 0.01)
SUVA (L/mg m)	2.6	3	3
DOC (mg/L)	2 (± 0.28)	1.1 (± 0.14)	10 (± 2.8)
TOC (mg/L)	2.2 (± 0.14)	1.2 (± 0.14)	–a
Total Coliform (MPN/100 ml)	5300 (± 1120)	Lower than 1	9500 (± 1625)
Fecal Coliform (MPN/100 ml)	1600 (± 256)	Lower than 1	2900 (± 414)
HPC (CFU/ml)	2550 (± 346)	50 (± 6)	4500 (± 525)

a Because of very high turbidity and particulate matter this parameter was not analysed.

**Table 2:** Quality of treated SFBW with primary sedimentation, coagulation and hybrid coagulation-UF process.

Parameter	Settled SFBW	Treated SFBW with PAFCl	Treated SFBW with FeCl_3_	PAFCl-UF output	FeCl_3_-UF output
Turbidity (NTU)	130 (± 1.6)	2.2 (± 0.28)	3.2 (± 0.3)	0.1 ≤	0.1 ≤
Color (Pt. Co. units)	30 (± 1.4)	3 (± 1.4)	4 (± 1.4)	0	0
EC(μs/cm)	330 (± 2.1)	339 (± 1.41)	352 (± 1.6)	339 (± 1.4)	352 (± 1.6)
TDS (mg/L)	165 (± 2.12)	167.3 (± 0.4)	175 (± 1.2)	167 (± 1.6)	175 (± 1.2)
pH	8.3 (± 0.08)	8.2 (± 0.2)	7.3 (± 0.3)	8.1 (± 0.1)	7.1 (± 0.14)
Alkalinity (mg/L CaCO_3_)	145 (± 2.1)	140 (± 1.4)	126 (± 2.1)	138 (± 1.2)	125 (± 1.6)
Sludge volume (ml/L)	3 (± 1.4)	5.1 (± 0.32)	7.2 (± 0.28)	Negligible	Negligible
Iron (mg/L)	0.35 (± 0.06)	0.03(± 0.002)	0.16 (± 0.01)	0	0
Aluminium (mg/L)	0.25 (± 0.04)	0.04 (± 0.003)	0.045(± 0.002)	0.035 (± 0.028)	0.027 (± 0.03)
Arsenic (µ/L)	0	0	0	0	0
Lead (µ/L)	14 (± 1)	8 (± 0.8)	11(± 0.92)	3 (± 0.2)	6 (± 0.5)
Cadmium (µ/L)	0. 61(± 0.08)	0.3 (± 0.06)	0.27 (± 0.05)	0.19 (± 0.04)	0.21 (± 0.03)
UVA_254nm_ (cm^−1^)	0.12 (± 0.02)	0.05 (± 0.01)	0.052 (± 0.014)	0.032 (± 0.01)	0.035 (± 0.01)
SUVA (L/mg m)	2.7	2.5	2.4	2.28	2.18
DOC (mg/L)	4.4 (± 0.28)	2 (± 0.28)	2.1 (± 0.3)	1.4 (± 0.1)	1.5 (± 0.11)
TOC (mg/L)	–a	2.3 (± 0.28)	2.47 (± 0.3)	1.7 (± 0.14)	1.8 (± 0.14)
Total Coliform (MPN/100 ml)	8500 (± 1414)	695 (± 77)	1075 (± 76)	Negative	Negative
Fecal Coliform (MPN/100 ml)	3050 (± 495)	585 (± 77)	920 (± 395)	Negative	Negative
HPC (CFU/ml)	3600 (± 565)	556 (± 62)	832 (± 181)	265 (± 35)	350 (± 42)

a Because of very high turbidity and particulate matter this parameter was not analysed.

**Table 3:** Mixing of settled SFBW with raw water that entered to Isfahan's WTP.

**Parameter**	**Mixing settled SFBW with raw water entered to WTP**
Turbidity (NTU)	9.8
Color (Pt. Co. units)	11.4
EC(μs/cm)	332
TDS (mg/L)	165
pH	8.22
Alkalinity (mg/L CaCO_3_)	132.3
Sludge volume (ml/L)	0.07
Iron (mg/L)	0.105
Aluminium (mg/L)	0.04
Arsenic (µ/L)	0
Lead (µ/L)	2.28
Cadmium (µ/L)	0.43
UVA_254nm_ (cm^−1^)	0.053
DOC (mg/L)	2.05
TOC (mg/L)	–a
Total Coliform (MPN/100 ml)	6986
Fecal Coliform (MPN/100 ml)	2512
HPC (CFU/ml)	885

a Because of very high turbidity and particulate matter this parameter was not analysed.

## 2 Experimental design, materials and methods

### 2.1 Quality, quantity and characteristics of raw SFBW

Isfahan water treatment plant treats 12 m^3^/s of water by coagulation, flocculation, sedimentation and rapid sand filtration processes. Produced backwash water from 48 filter units in the plant considered as a waste. Generated SFBW was about 24,000 m^3^/d.

#### 2.1.1 Experiment protocol

In this study SFBW was treated by continues processes including primary sedimentation, coagulation, flocculation, secondary sedimentation and UF process. Entrance flow for all sections of the pilot, except UF membrane was 10 l/h. Also, hydraulic retention time (HRT) was 60, 6, 48 and 192 minu, respectively (Fig. 1). According to our previous study, coagulation with PAFCl and FeCl_3_ was conducted at pH 8.3 [1]. Both PAFCl and FeCl_3_ used as coagulants (Fig. 2, Fig. 3). A pre-determined dosage of PAFCl (10 mg/L) and FeCl_3_ (30 mg/L) was continuously and individually added into the rapid mixer basin (mixing speed: 80 rpm, HRT: 6 min). Then coagulated water passed through two-flocculation tanks with a mixing intensity of 40 rpm. After that, the effluent was introduced to a secondary sedimentation basin, and then directed to a UF membrane module. The UF module filtration was 8 L m^−^^2^ h^−^^1^ at a trans-membrane pressure of 300 Pa. It was operated in a cycle of 60 min filtration and 1 min backwashing [2], [3], [4], [5]. The importance of such treatment processes for SFBW is that in case there are some concentrations of pollutants being accumulated in the SFBW they will be removed to much lower concentrations [7], [8], [9]. Data of this article attained from experimental work and all experiments were conducted according to the standard method for the examination of water and wastewater. Total organic carbon (TOC) was analyzed by Phoenix 8000 system. Turbidity, UV_254_, true color, Total dissolved solid (TDS), Electrical Conductivity, and pH were measured by TN-100 (EUTECH) Turbidimeter, DR 5000-HACH LANGE, EC meter SENSION5 (HACH-LANGE, Germany), and pH-meter model CG 824, respectively. Dissolved organic carbon (DOC), UV254, and the true color were analyzed after filtration through a 0.45 μm membrane. Fe, Pb, As, and Cd were analyzed by inductively coupled plasma (ICP).
