# Supplementary material for: Dataset on the spent filter backwash water treatment by sedimentation, coagulation and ultra filtration
Source: Data Brief. 2017 Nov 2;15:916–21. doi: 10.1016/j.dib.2017.10.062 (PMC5683744; doi:10.1016/j.dib.2017.10.062)
Supplement: Supplementary file 1 — Transparency document [file mmc1.doc]

**COVERING LETTER**

| **Article Title**  Dataset on the spent filter backwash water treatment by sedimentation, coagulation and ultra filtration |
| --- |
| **the name of the journal:**  **DATA IN BRIEF** |
| **the full names of all authors, clearly indicating the corresponding author**   | **1- Mokhtar Mahdavi** |  | **1- Afshin Ebrahimi** | | --- | --- | --- | | **2- Hossein Azarpira** |  | **3-** Hamid Reza Tashauoei | | **4- Amir Hossein Mahvi** |  | **-** |   1- Assistant Professor of Environmental Health Engineering Department, Saveh University of Medical Sciences, Social Determinants of Health Research Center, Saveh, Iran. [ShamaLL6@yahoo.com](mailto:ShamaLL6@yahoo.com).  2- Department of Environmental Health Engineering, Environment Research Center, Research Institute for Primordial Prevention of Non Communicable Disease, Isfahan University of Medical Sciences, Isfahan, Iran. [a_ebrahimi@hlth.mui.ac.ir](mailto:a_ebrahimi@hlth.mui.ac.ir), +983117783247  3- Department of Environmental Health Engineering, School of Health, Islamic Azad University Tehran Medical Branch, Tehran, Iran. [h.tashauoei@nww.ir](mailto:h.tashauoei@nww.ir) +989138759208  4- Center for Solid Waste Research, Institute for Environmental Research, Tehran University of Medical Science, Tehran, Iran. [ahmahvi@yahoo.com](mailto:ahmahvi@yahoo.com).  * Corresponding author, Amir Hossein Mahvi- Center for Solid Waste Research, Institute for Environmental Research, Tehran University of Medical Science, Tehran, Iran. [ahmahvi@yahoo.com](mailto:ahmahvi@yahoo.com). +989123211827 |
| **Abstract**  During operation of most water treatment plants, spent filter backwash water (SFBW) is generated, which accounts about 2 to 10% of the total plant production. By increasing world population and water shortage in many countries, SFBW can be used as a permanent water source until the water treatment plant is working. This data article reports the practical method being used for water reuse from SFBW through different method including pre-sedimentation, coagulation & flocculation, second clarification, ultra filtration (UF) and returned settled SFBW to the beginning of water treatment plant (WTP). Also, two coagulants of polyaluminum ferric chloride (PAFCl) and ferric chloride (FeCl_3_) were investigated with respect to their performance on treated SFBW quality. Samples were collected from Isfahan's WTP in Iran during spring and summer season. The acquired data indicated that drinkable water can be produced form SFBW by applying hybrid coagulation-UF process (especially when PAFCl used as coagulant). |
| **Key Words**  Spent filter backwash water, water treatment, coagulation, ultra-filtration |

| Name and address of corresponding author  Amir Hossein Mahvi- Center for Solid Waste Research, Institute for Environmental Research, Tehran University of Medical Science, Tehran, Iran. [ahmahvi@yahoo.com](mailto:ahmahvi@yahoo.com). +989123211827 | |
| --- | --- |
| Telephone #  +989123211827 | Fax # |
| Email [ahmahvi@yahoo.com](mailto:ahmahvi@yahoo.com) | |

I affirm that the manuscript has been prepared in accordance with International Journal of **DATA IN BRIEF**instructions to authors and the content of this manuscript, or a major portion thereof, has not been published in a referred journal nor being submitted for publication elsewhere.

Signature of Corresponding Author Date

**21/08/ 2017**

**
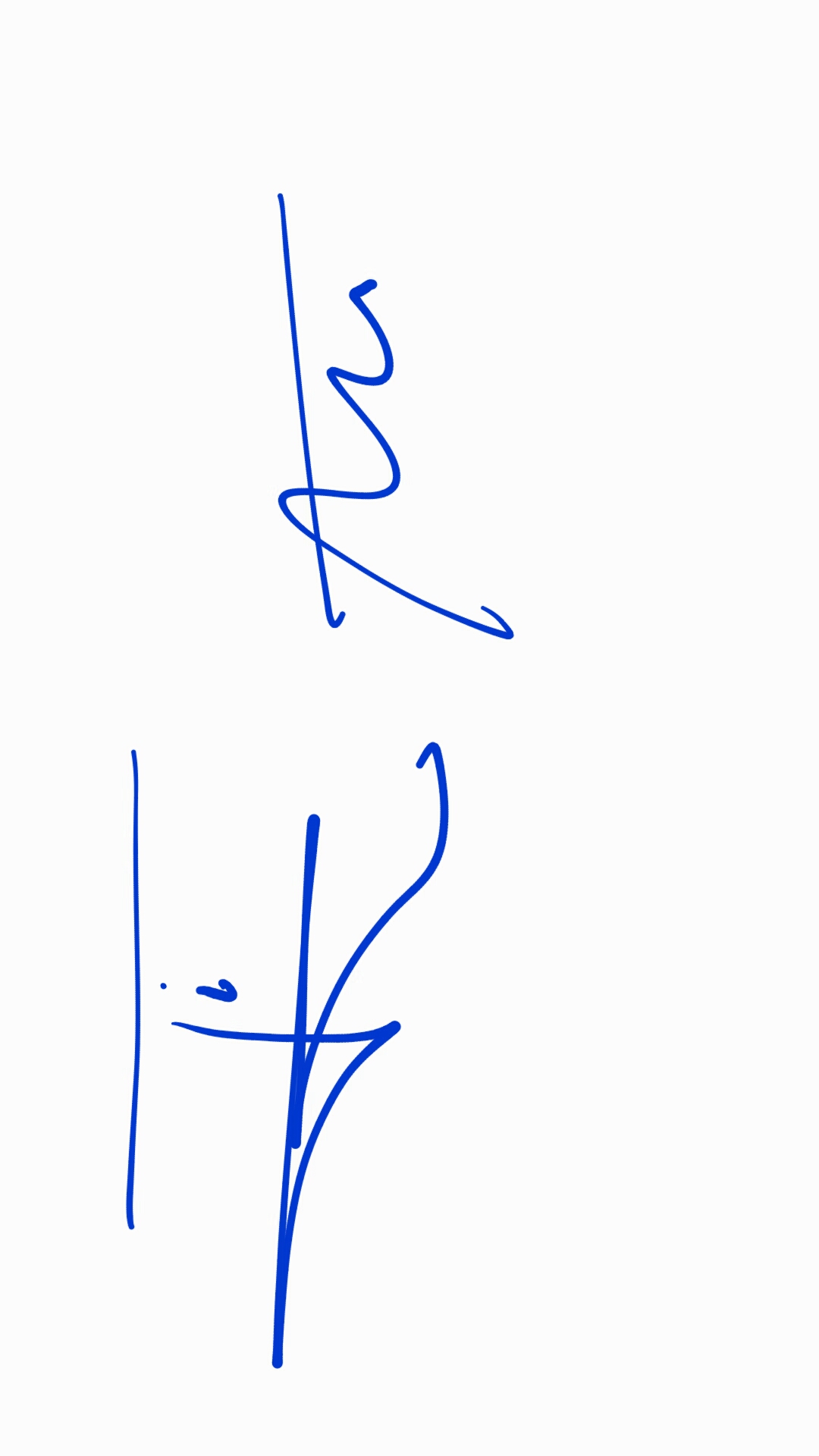
**
